# A Controlled Study of Tuberculosis Diagnosis in HIV-Infected and Uninfected Children in Peru

**DOI:** 10.1371/journal.pone.0120915

**Published:** 2015-04-30

**Authors:** Richard A. Oberhelman, Giselle Soto-Castellares, Robert H. Gilman, Maria E. Castillo, Lenka Kolevic, Trinidad Delpino, Mayuko Saito, Eduardo Salazar-Lindo, Eduardo Negron, Sonia Montenegro, V. Alberto Laguna-Torres, Paola Maurtua-Neumann, Sumona Datta, Carlton A. Evans

**Affiliations:** 1 Tulane School of Public Health and Tropical Medicine, New Orleans, Louisiana, United States of America; 2 Asociación Benéfica Proyectos en Informatica, Salud, Medicina, y Agricultura (PRISMA), Lima, Peru; 3 US Naval Medical Research Unit Six, Lima, Peru; 4 Department of Microbiology, Faculty of Sciences and Philosophy, Universidad Peruana Cayetano Heredia, Lima, Peru; 5 Department of International Health, Johns Hopkins Bloomberg School of Public Health, Baltimore, Maryland, United States of America; 6 Infectious Diseases Service, Instituto Nacional de Salud del Niño, Lima, Peru; 7 Department of Pediatrics, Faculty of Medicine, Universidad Peruana Cayetano Heredia, Lima, Peru; 8 Department of Pediatrics, Hospital Nacional Cayetano Heredia, Lima, Peru; 9 Universidad de Concepción, Concepción, Chile; 10 Department of Pediatrics, Tulane University School of Medicine, New Orleans, Louisiana, United States of America; 11 Infectious Diseases & Immunity, Imperial College London, and Wellcome Trust Imperial College Centre for Global Health Research, London, United Kingdom; 12 IFHAD: Innovation For Health And Development, London, United Kingdom; Hopital Raymond Poincare - Universite Versailles St. Quentin, FRANCE

## Abstract

**Background:**

Diagnosing tuberculosis in children is challenging because specimens are difficult to obtain and contain low tuberculosis concentrations, especially with HIV-coinfection. Few studies included well-controls so test specificities are poorly defined. We studied tuberculosis diagnosis in 525 children with and without HIV-infection.

**Methods and Findings:**

‘Cases’ were children with suspected pulmonary tuberculosis (n = 209 HIV-negative; n = 81 HIV-positive) and asymptomatic ‘well-control’ children (n = 200 HIV-negative; n = 35 HIV-positive). Specimens (n = 2422) were gastric aspirates, nasopharyngeal aspirates and stools analyzed by a total of 9688 tests.

All specimens were tested with an in-house hemi-nested IS6110 PCR that took <24 hours. False-positive PCR in well-controls were more frequent in HIV-infection (P≤0.01): 17% (6/35) HIV-positive well-controls versus 5.5% (11/200) HIV-negative well-controls; caused by 6.7% (7/104) versus 1.8% (11/599) of their specimens, respectively. 6.7% (116/1719) specimens from 25% (72/290) cases were PCR-positive, similar (P>0.2) for HIV-positive versus HIV-negative cases.

All specimens were also tested with auramine acid-fast microscopy, microscopic-observation drug-susceptibility (MODS) liquid culture, and Lowenstein-Jensen solid culture that took ≤6 weeks and had 100% specificity (all 2112 tests on 704 specimens from 235 well-controls were negative). Microscopy-positivity was rare (0.21%, 5/2422 specimens) and all microscopy-positive specimens were culture-positive. Culture-positivity was less frequent (P≤0.01) in HIV-infection: 1.2% (1/81) HIV-positive cases versus 11% (22/209) HIV-negative cases; caused by 0.42% (2/481) versus 4.7% (58/1235) of their specimens, respectively.

**Conclusions:**

In HIV-positive children with suspected tuberculosis, diagnostic yield was so low that 1458 microscopy and culture tests were done per case confirmed and even in children with culture-proven tuberculosis most tests and specimens were false-negative; whereas PCR was so prone to false-positives that PCR-positivity was as likely in specimens from well-controls as suspected-tuberculosis cases. This demonstrates the importance of control participants in diagnostic test evaluation and that even extensive laboratory testing only rarely contributed to the care of children with suspected TB.

**Trial Registration:**

This study did not meet Peruvian and some other international criteria for a clinical trial but was registered with the ClinicalTrials.gov registry: ClinicalTrials.gov NCT00054769

## Introduction

HIV-infection is associated with tuberculosis (TB) disease, atypical clinical and x-ray characteristics and low mycobacterial concentrations that delay TB diagnosis, potentially increasing morbidity, mortality and TB transmission [1,2]. Challenges diagnosing pulmonary TB are similar and synergistic for patients with Human Immunodeficiency Virus (HIV) co-infection and children [3], are confounded by difficulty obtaining sputum from children and are worsened by the inadequacy of laboratory tests. Sensitive liquid culture is generally costly, while traditional solid culture methods are slower and less sensitive. Even with optimal resources, laboratory confirmation of presumed pediatric TB diagnoses is achieved in only a minority of cases [4–7]. Consequently, clinicians usually rely on their clinical impression and/or clinical scoring systems that are poorly validated [8–10]. We still depend heavily on tools that have been available and inadequate for more than a century to presumptively diagnose TB disease in children: tuberculin skin test, chest x-ray, history and physical examination [11].

While TB diagnosis in adults co-infected with HIV has been widely studied, optimal approaches, specimens and tests are less well characterized in pediatric patients and are undermined by the lack of a gold standard for determining which children have TB. Patient series from South Africa reported HIV co-infection in 32% of children with culture-confirmed TB [12] and similar culture positivity of induced sputum specimens in HIV-positive and HIV-negative children [13]. However, other data suggest that diagnostic yields in pediatric patients with suspected pulmonary TB were lower in HIV-infected than HIV-negative children [14–15] and most studies have focused on high-burden countries in southern Africa. The epidemiology of TB and HIV are very different in Latin America versus southern Africa, with markedly lower overall HIV seroprevalence among new cases (for example, <5% in Peru versus >50% in southern Africa).

In children, especially those living with HIV-infection, even a low percentage of false-positive laboratory results may disproportionately affect test interpretation because they may have comparable frequency to true-positives. This has been the subject of little research and few evaluations of pediatric TB diagnosis included control children who do not have suspected TB. Previous studies evaluating pediatric TB diagnostic approaches, specimens and tests have usually assumed that any positive laboratory result confirms TB and there is a lack of knowledge concerning false-positivity rates and how these may be affected by HIV co-infection.

Recently the disposable cartridge-based GeneXpert MTB/RIF commercial assay has been approved for TB diagnosis with subsidized pricing available to selected health-care providers in selected countries. However, in most resource-constrained settings subsidies are unavailable and costs per test are typically $60-$100. This is too expensive for widespread use in these settings because TB principally affects socioeconomically disadvantaged groups. In contrast, costs per test for in-house PCR TB diagnostic assays are <$5 per specimen and this affordability may in some settings balance the procedural and logistical challenges of using in-house PCR assays [16–17].

We have published a study of diagnostic approaches for TB in HIV-negative Peruvian children with suspected pulmonary TB and well-controls that defined the microbiological contribution of specimens and tests, including a low-cost in-house polymerase chain reaction (PCR) technique [18]. In this current report we assess the performance of these laboratory tests in concurrently tested HIV-infected children, and demonstrate the contrast with previously published results for HIV-negative children.

## Materials and Methods

### Ethics statement

Participants were enrolled by written parental informed consent and for children ≥7 years additional written child participant assent. The project was approved by the institutional review boards of Tulane Medical Center, Johns Hopkins Bloomberg School of Public Health, Asociación Benéfica PRISMA, the US Naval Medical Research Center (Bethesda, MD), Hospital Nacional Cayetano Heredia, and the Instituto Nacional de Salud del Niño of Lima, Peru. Empiric treatment for TB was administered according to World Health Organization and Peruvian Ministry of Health guidelines. Therapeutic decisions including hospitalization and treatment were determined by local physicians who were independent from this study.

**HIV-positive cases and well-controls** are reported here for the first time compared with new analysis of our previously reported data for HIV-negative participants [18]. Eleven italicized data points for HIV-negative participants in Table 1 and four data points in Table 2 are repeated from our previous publication [18] for comparison; all other data and analyses are unique to this manuscript.

**Table 1:** Study population demographic and clinical features.

		116 HIV-positive children			409 HIV-negative children			P-value for HIV-negative vs HIV-positive children	
		81 TB cases with ST>3	35 well-controls	P-value for cases vs well-controls	209 TB cases with ST>4	200 well-controls	P-value for cases vs well-controls	290 cases	235 well-controls
*Years old*	Median	3	5		*3*	*4*	*0*.*08*	0.08	0.3
	IQR	1–6	3–7		*1–7*	*2–7*			
*Gender*	% male	49	57	0.4	*50*	*54*	*0*.*5*	0.9	0.7
	95%CI	38–61	39–74		*43–57*	*47–61*			
*Weight-for-age*	Median	74	89	<0.001	*96*	*94*	*NS*	<0.001	0.01
	IQR	65–88	80–95		*86–106*	*87–103*			
*Low income*	%	76	67	0.4	*47*	*78*	<0.001	<0.001	0.2
	95%CI	64–86	45–84		*40–54*	*71–83*			
*Cough*	%	96	5.7	<0.001	*75*	*27*	<0.001	<0.001	0.006
	95%CI	92–100	0–13		*69–81*	*21–33*			
*Highly probable TB (ST score >6)*	%	21	0	<0.001	71	0	<0.001	<0.001	ND
	95%CI	12–30	0–9.9		65–77	0–1.8			
*Hemoptysis*	%	6.2	0	<0.001	3.4	1.0	0.1	0.3	0.6
	95%CI	1.0–11	0–9.9		1.0–5.8	0–2.4			
*Fever*	%	54	5.7	<0.001	28	15	0.0007	<0.001	0.2
	95%CI	43–65	0–13		22–34	9.6–19			
*Anorexia*	%	40	2.9	<0.001	40	23	0.0002	0.9	0.007
	95%CI	30–51	0–8.4		33–46	17–28			
*Recent weight loss*	%	43	2.9	<0.001	28	11	<0.001	0.016	0.1
	95%CI	32–54	0–8.4		22–34	6.5–15			
*Diarrhea*	%	44	11	0.0008	13	17	0.3	<0.001	0.4
	95%CI	33–55	0.89–22		8.8–18	12–22			
*Vomiting*	%	26	2.9	0.004	13	6.5	0.03	0.008	0.4
	95%CI	16–35	0–8.4		8.4–17	3.1–9.9			
*CD4 count*	%	365	660	0.01	ND	ND	ND	NA	NA
	95%CI	133–749	130–1012		ND	ND			
Abnormal chest x-ray *+	%	99	ND	ND	*83*	ND	ND	<0.001	ND
	95%CI	96–100	ND		*77–88*	ND			
*Alveolar infiltrate +*	%	23	ND	ND	5.2	ND	ND	<0.001	ND
	95%CI	13–32	ND		1.9–8.5	ND			
*Interstitial infiltrate +*	%	84	ND	ND	54	ND	ND	<0.001	ND
	95%CI	76–92	ND		47–62	ND			
*Nodular infiltrate +*	%	6.7	ND	ND	4.1	ND	ND	0.2	ND
	95%CI	1.0–12	ND		1.1–7.0	ND			
*Calcification +*	%	0.0	ND	ND	1.1	ND	ND	0.3	ND
	95%CI	0–4.6	ND		0.1–4.0	ND			
*Pleural thickening +*	%	1.3	ND	ND	3.3	ND	ND	0.3	ND
	95%CI	0.3–6.9	ND		1.2–7.1	ND			
*Cavity/cavities +*	%	0	ND	ND	1.7	ND	ND	0.3	ND
	95%CI	0–4.6	ND		0.3–4.8	ND			
*Lymphadenopathy +*	%	3.8	ND	ND	7.2	ND	ND	0.3	ND
	95%CI	0.8–11	ND		3.4–11	ND			
*Hilar swelling +*	%	34	ND	ND	44	ND	ND	0.2	ND
	95%CI	24–46	ND		36–51	ND			
*Primary complex +*	%	19	ND	ND	19	ND	ND	1.0	ND
	95%CI	11–30	ND		14–26	ND			

+ All cases had x-rays that defined their ST score. Detailed research x-ray scoring was only available for 93% (75/81) HIV-positive cases and 83% (173/209) HIV-negative cases (see Results).

*no participants had miliary or bullous chest x-ray findings so these findings are not listed in the table

Note: ND = not done because controls did not have these symptoms and did not have chest x-rays; NS = not significant (P>0.05); NA = not applicable as data was not collected; TB = tuberculosis; ST = the Stegen-Toledo score assessing the likelihood of TB disease (see Methods); low income = below the monthly minimum wage in Peru that was 400 Soles (approximately $115 USD monthly) at the time of the study; weight-for-age was based on Centers for Diseases Control growth standards.

**Table 2:** PCR and culture results.

	Test	Positive specimens	116 HIV-positive children									409 HIV-negative children									HIV-negative vs HIV-positive	
Children			81 TB cases with ST>3 6 specimens per child				35 well controls with 3 specimens per child				TB cases vs well-controls	209 TB cases with ST>4 6 specimens per child				200 well controls with 3 specimens per child				TB cases vs well-controls	290 TB cases	235 well controls
			%	95%CI	n	N	%	95%CI	n	N	P-value	%	95%CI	n	N	%	95% CI	n	N	P-value	P-value	P-value
	PCR	Any	22%	13–31	18	81	17%	4.7–30	6	35	NA	*26%*	*20–32*	*54*	*209*	*5*.*5%*	*2*.*3–8*.*7*	*11*	*200*	*NA*	0.5	0.01
	Microscopy & culture	Any	1.2%	0.0–3.6	1	81	0.0%	0.0–9.9	0	35	NA	*11%*	*6*.*3–15*	*22*	*209*	*0*.*0%*	*0*.*0–1*.*9*	*0*	*200*	*NA*	0.01	1.0
	P-value PCR vs microscopy & culture		<0.0001				0.01				NA	*<0*.*0001*				*0*.*0008*				*NA*	NA	NA
Specimens	PCR	NPA	4.9%		8	162	8.6%	0.0–18	3	35	0.4	7.0%	4.5–9.4	29	415	2.0%	.05–3.9	4	201	0.01	0.4	0.03
		GA	4.3%	1.2–7.5	7	162	ND	ND	ND	ND	ND	9.9%	7.0–13	41	416	ND	ND	ND	ND	NA	0.03	NA
		Stool	7.6%	3.5–12	12	157	5.8%	.28–11	4	69	0.6	4.7%	2.6–6.7	19	407	1.8%	.47–3.1	7	398	0.02	0.2	0.04
		NPA+GA+ Stool	5.6%	3.6–7.8	27	481	6.7%	1.9–12	7	104	0.7	7.2%	5.8–8.6	89	1238	1.8%	.76–2.9	11	599	<0.001	0.2	0.003
	Microscopy & culture	NPA	0.0%	0.0–2.3	0	162	0.0%	0.0–9.9	0	35	1.0	4.8%	2.8–6.9	20	414	0.0%	*0*.*0–1*.*9*	0	201	<0.001	0.004	1.0
		GA	1.2%	0.0–2.9	2	162	ND	ND	ND	ND	ND	8.0%	5.3–11	33	415	ND	ND	ND	ND	NA	0.002	NA
		Stool	0.0%	0.0–2.4	0	157	0.0%	0.0–5.3	0	69	1.0	1.2%	.16–2.3	5	406	0.0%	0.0–1.0	0	399	0.03	0.2	1.0
		NPA+GA+ Stool	0.42%	0.0–1.0	2	481	0.0%	0.0–3.6	0	104	0.5	4.7%	3.5–5.9	58	1235	0.0%	0.0–0.64	0	600	<0.001	<0.001	1.0
	P-value PCR vs microscopy & culture		<0.001				0.01				NA	0.01				0.001				NA	NA	NA

The percentage and number of children and specimens from children with at least one positive PCR or culture.

The Stegen-Toledo cut-off for inclusion in this analysis differed between HIV-positive and HIV-negative children (see Methods).

Note: TB = tuberculosis; NPA = nasopharyngeal aspirate; GA = gastric aspirate; NA = not applicable; ND = not done; ST = the Stegen-Toledo score assessing the likelihood of TB disease (see Methods).

**Fig 1:**
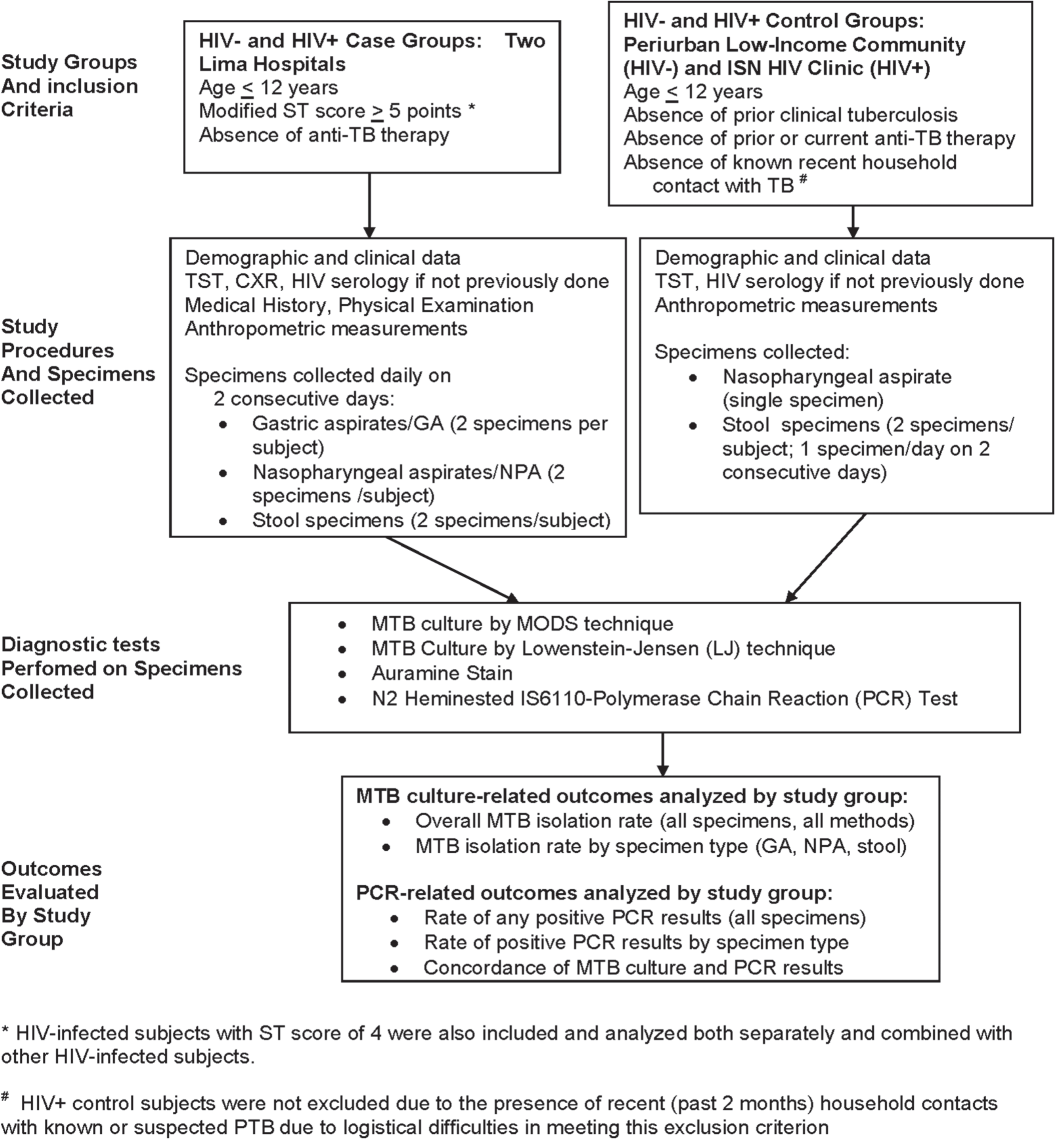
Study flow chart.

Study design is shown in Fig 1. **Recruitment** took place continuously 2002–2007 and all participants were age <13 years.

**Participant classification** used the criteria of Stegen and Jones [19] (“Jones Score” in Africa) for pediatric TB diagnosis as revised by Toledo. This “Stegen-Toledo” (ST) score is used throughout Latin America [20] and is graded as the sum of: 2 points for known tuberculosis contact in the last 2 years; 2 points for x-ray suggestive of tuberculosis; 2 points for cough for >2 weeks or other suggestive clinical characteristic; 4 points for tuberculous granuloma; and 4 points for positive tuberculin skin test (≥5 mm induration in HIV-positive children; ≥10 mm for HIV-negative children as was local practice). Usually the Stegen-Toledo score also includes 7 points for a positive tuberculosis culture, but this was excluded because culture results were a primary outcome of our study and were not available at the time of enrollment. The Stegen-Toledo score is interpreted as: unlikely tuberculosis (0 or 2 points); suspicious for tuberculosis (4 points); probable tuberculosis (6 points); or highly probable tuberculosis (>6 points).

**Participants** were:

-“**cases**” with clinical evidence suggestive of pulmonary TB at the Instituto de Salud del Niño and the Hospital Nacional Cayetano Heredia; and
-“**well-controls**” who were asymptomatic and enrolled concurrently from households in a low-income shantytown, all in Lima, Peru as described [18].

**Inclusion criteria for cases** were based on the Stegen-Toledo score. Because there is no gold standard for pediatric TB diagnosis, we defined cases as children with suspected TB, i.e. a Stegen-Toledo score >4 (i.e. probable or highly-probable TB). HIV-positive cases with Stegen-Toledo score >3 (i.e. also including score = 4 indicating suspicious for TB) were included as cases for the primary analysis because HIV co-infected children are usually treated empirically if the clinical suspicion of TB disease is lower than for HIV-negative children. A supplementary analysis considered the same inclusion criteria for HIV-negative and HIV-positive cases i.e. only cases with Stegen-Toledo score >4.

**Inclusion criteria for well-controls** were 1) absence of cough, fever, or evidence of pulmonary disease at the time of enrollment; and 2) no prior diagnosis of TB disease. We aimed to recruit only well-control children who were each age and sex matched to cases and had no known or suspected household contact with pulmonary TB in the past 2 years. These criteria could not be applied perfectly for HIV-positive well-controls because HIV-infection is uncommon in Peru, which impeded recruitment.

**Screening for HIV infection** by duplicate commercial antibody assays was requested for all participants. Children <18 months old also had HIV PCR. Participants who declined HIV testing and had no known HIV exposure or clinical evidence of HIV/AIDS were included and classified as HIV-negative because in Lima HIV infects <0.5% adults and <0.1% children aged <12 years.

**Demographic data** were collected from all participants. Cases had medical history, physical examination and a chest x-ray read by a research pediatric radiologist who used a standardized reading scale and who was blinded to all clinical data. Presence of symptoms during the 15 days prior to enrollment were recorded.

**Specimens** were all collected before any anti-TB therapy and we aimed to collect 1 specimen of each type per day. All specimens were collected within 5 days of enrollment.

**Specimens from cases** were 2 of each of the following specimens: stool specimens; nasopharyngeal aspirates (NPA) collected by inserting a soft flexible nasopharyngeal tube into the nasopharynx, lavaging with 5 ml sterile physiological saline solution, and aspirating with an electrical suction device or hand-held aspirator; and gastric aspirates collected early mornings (6–7 am) following an overnight fast by brief (<10 minute) nasogastric intubation (if aspiration was unsuccessful then gastric lavage was done but the frequency of this was not recorded).

**Specimens from well-controls** were 2 stool specimens and 1 nasopharyngeal aspirate. The gastric aspirates and repeat nasopharyngeal aspirates were omitted to increase acceptability.

### Laboratory tests

All specimens were transported to the laboratory the same day as collection at 4°C without buffering or neutralization. Specimens were processed as described [18] by sodium hydroxide centrifuge decontamination followed by:

1. Auramine smear microscopy stain (smear test);
2. Lowenstein-Jensen solid culture [21–22];
3. Microscopic-observation drug-susceptibility (MODS) culture that in order to maximize diagnostic sensitivity was performed without using any of the diagnostic specimens for concurrent drug-susceptibility testing, as described previously [18, 21, 22]; and
4. hemi-nested IS6110-PCR procedure (“PCR”) [16, 18] that used standard precautions to prevent PCR products from contaminating specimens including separate restricted access rooms for specimen processing versus manipulation of PCR products. All PCR assays included positive and negative PCR controls that in all cases gave appropriate positive and negative results, respectively.

### Analysis

Chi-square and McNemar’s tests were used for categorical variables and two-tailed T-test or Wilcoxon rank sum test for continuous variables. Confidence intervals, numerators and denominators for key outcomes are stated in the text only when they are not presented in the tables and/or figures. Participants with any culture-positive specimen by any test were classified as culture-positive. To reduce the risk of failed contaminated cultures being misinterpreted, culture-negative cases were only included in the analysis if all specimens had at least 1 interpretable, un-contaminated Lowenstein-Jensen and MODS result. Participants with at least one PCR-positive specimen were classified as PCR-positive. Analysis was performed using STATA Version 11 (STATA Corp., College Station, TX) and EpiInfo (Version 6, Centers for Disease Control and Prevention; Atlanta, GA, USA) software and all data are calculated to 2 significant figures. The dataset for this study is included as supplemental information along with a legend defining specific variables (S1 Dataset). All laboratory techniques were performed by qualified biologists who were trained, supervised, used written standard operating procedures with internal quality control and were blinded to the results of all other tests, including HIV-status.

## Results

**Participants** constituted 525 children in 4 groups with sufficient data to meet our protocol criteria for inclusion in the analysis [18]:

209 HIV-negative cases;
81 HIV-positive cases;
200 HIV-negative well-controls; and
35 HIV-positive well-controls.

**Non-participants excluded from the analysis** (n = 54) were the following children enrolled in the study: 9 HIV-negative cases; 4 HIV-positive cases; 31 HIV-negative well-controls; and 10 HIV-positive well-controls. These exclusions were caused by: parents refusing study procedures (n = 12); loss to follow-up (n = 14); and missing data (n = 28).

**Participant characteristics** are shown in Table 1. The patient groups had similar characteristics except: HIV-positive well-controls were older; HIV-positive cases had lower weight-for-age; and fewer HIV-negative cases had low income than other groups. As expected, symptoms of illness reported during the last 15 days were more common in cases than in controls.

**HIV-**positive subjects received co-trimoxazole, but antiretroviral therapy for children did not begin to become available until a year after recruitment ended. Mean CD4 count of the 66 HIV-positive TB cases with this information (365) was significantly lower than the mean value for the 27 HIV-positive controls with CD4 data (660; P = 0.01). No HIV-positivity was newly diagnosed by this study and 82% of HIV-negative statuses were laboratory confirmed.

### X-rays (Table 1)

All cases had x-rays that defined their Stegen-Toledo score. Detailed research x-ray scoring data are shown in Table 1 and were only available for 93% (75/81) HIV-positive cases and 83% (173/209) HIV-negative cases.

**Fig 2:**
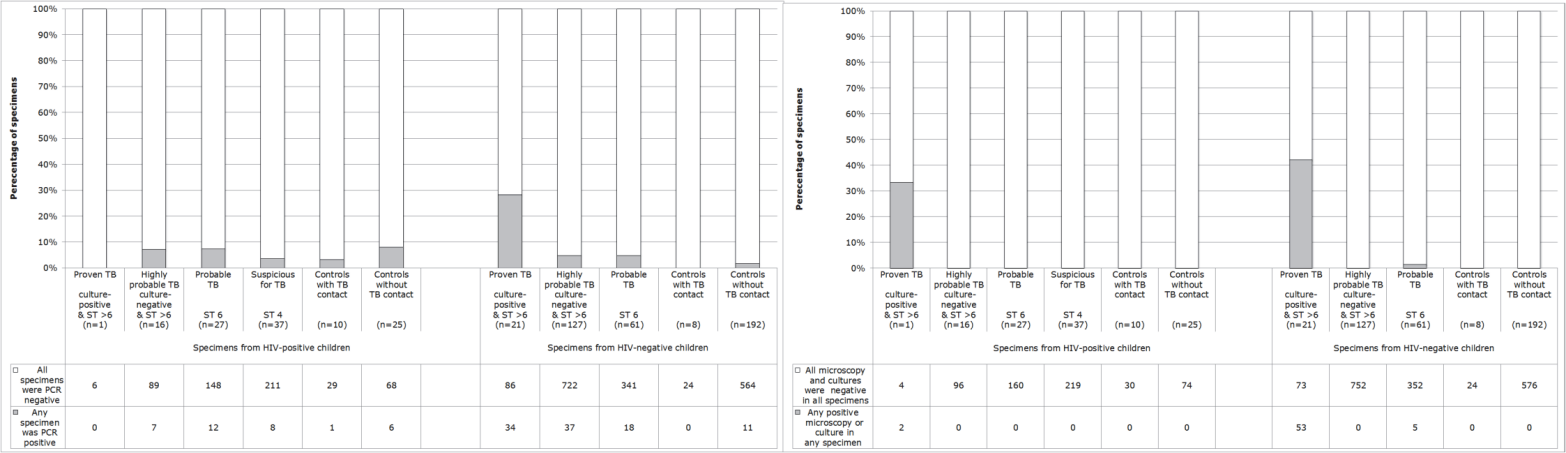
Tuberculosis (TB) test results for specimens from HIV-positive and HIV-negative case and well-control children. Data are analysed by assessment of probability of TB disease indicated by the Stegen-Toledo (ST) clinical score (see Methods). (A) TB PCR results. (B) Microscopy and culture test results: auramine microscopy, microscopic-observation drug-susceptibility (MODS) liquid culture, and Lowenstein Jensen solid culture.

**Stegen-Toledo scores** were lower for HIV-positive cases versus HIV-negative cases whether HIV-positive children with Stegen-Toledo score 4 (suspicions for TB) were included (in the primary analysis) or excluded (for the secondary analysis that used the same Stegen-Toledo inclusion criteria for HIV-positive as HIV-negative cases; Fig 2; both comparisons P<0.001). Similarly, several symptoms were more frequent in HIV-positive cases than HIV-negative (cough, fever, weight loss, diarrhea, vomiting, Table 1).

**Test results** in Table 2 do not compare case children versus well-control children because this would be invalid because cases each had 6 specimens tested (including 2 gastric aspirates that yielded most positives), whereas controls each had only 3 specimens tested (without gastric aspirates).

### False-positive PCR (Table 2)

18 specimens from well-controls were PCR positive, causing 17 well-controls to be PCR positive. No follow-up to determine clinical diagnoses, treatment, or treatment outcome were included in our research protocol because these would be influenced by the test results that were being assessed by this study. However, for ethical reasons only these 17 PCR-positive well-controls were followed up at least 3 monthly for 1 year including at least 1 chest x-ray during this follow-up that was 100% completed in all cases and revealed no evidence of TB disease. PCR-positivity in well-controls tended to be more common in specimens from well-controls without household TB contact than with TB contact (Fig 2). Because this PCR-positivity in well-controls was not associated with subsequent TB disease or prior TB exposure, these PCR-positive results in well-controls are henceforth termed false-positives. False-positive PCR occurred similarly frequently in all specimen types (Table 2) and were dispersed chronologically and had similar frequency early and late in the project (data not shown).

**PCR specificity (Table 2)** per specimen in well-controls was 93% (95%CI 88–98, 97/104) for specimens from HIV-positive participants, lower than 98% (95%CI 97–99, 588/599) for specimens from HIV-negative participants (because false-positive PCR were more frequent in 6.7% specimens from well-controls who were HIV-positive versus 1.8% HIV-negatives, P = 0.0003). Consequently, PCR specificity per well-control child was 83% (95%CI 70–95, 29/35) for HIV-positive participants, less than 95% (95%CI 91–98, 189/200) for HIV-negative children (because well-controls were more often PCR false-positive in 17% HIV-positives versus 5.5% HIV-negatives, P = 0.01).

**PCR sensitivity (Fig 2)** in culture-proven TB was 0.0% in HIV-positive children (because PCR were false-negative for all 6/6 specimens from culture-proven HIV-positive cases) and 28% (95%CI 20–36, 34/120, P = 0.1) in HIV-negative children (because 86/120 specimens from culture-proven HIV-negative cases were false-negative). Thus overall PCR sensitivity in all culture-proven TB was 27% (95%CI 19–36% because 92/126 specimens from children with culture-proven TB were PCR false-negative). Cases without culture-proven TB cannot be used to assess PCR sensitivity because it is unknown how many had TB disease versus alternative diagnoses.

### PCR yield (Table 2)

6.7% (95%CI 5.6–8.0, 116/1719) specimens from cases were PCR-positive causing 25% (95%CI 5.6–8.0, 72/290) cases to be PCR-positive. PCR-positivity was similarly frequent (P = 0.2) for 5.6% specimens from HIV-positive cases versus 7.2% HIV-negatives. Consequently, PCR-positivity was similarly frequent (P = 0.5) for 22% HIV-positive cases versus 26% HIV-negatives.

### PCR utility

Specimens from HIV-positive participants were more likely to be PCR-positive in 6.7% well-controls than: 5.6% cases; and 0.0% cases with culture-proven TB (both P>0.5). Thus, this in-house PCR did not have clinical utility for TB diagnosis in HIV-positive children. Specimens from HIV-negative participants were 4.0-times more likely (P<0.001) to be PCR-positive in 7.2% cases than 1.8% well-controls, so these results may have had some clinical utility in HIV-negative children in this study [18].^18^

**Microscopy and culture specificity (Table 2)** was 100% because all results were negative for 2112 tests in 704 specimens from 235 well-controls.

**Microscopy sensitivity (Fig 2)** in culture-proven TB was 0.0% in HIV-positive children (because microscopy was false-negative in all 6/6 specimens from culture-proven HIV-positive cases) and 4.0% (95%CI 0.56–7.4, 5/126, P = 0.6) in HIV-negative children (because 121/126 specimens from culture-proven HIV-negative cases were microscopy false-negative). Thus overall microscopy sensitivity in all culture-proven TB was 4% (95%CI 1.2–8.6, because 121/132 specimens from children with culture-proven TB were microscopy false-negative).

### Microscopy utility

All microscopy-positive specimens were culture-positive so microscopy contributed no additional diagnoses. Microscopy-positivity was rare, occurring in only 0.21%, (95%CI 0.067–0.5, 5/2422) of specimens. However, microscopy is a low-cost test that provides same-day results and is considered an integrated part of culture-testing protocols in many laboratories so had some utility in this study.

**Microscopy and culture sensitivity (Fig 2)** in culture-positive participants was 42% (because 58% (95%CI 49–67, 73/126) specimens from culture-proven TB cases were microscopy and culture false-negative). None of the culture-positive cases had multi-drug resistant TB. Microscopy and culture sensitivity was 33% (95%CI 0.0–71, 2/6) in HIV-positive culture-positive participants and 42% (95%CI 33–51, 53/126, p = 0.7) in HIV-negatives. Cases without culture-proven TB cannot be used to assess sensitivity because it is unknown how many had TB disease versus alternative diagnoses.

### Microscopy and culture yield (Table 2)

Microscopy or culture were positive in 0.42% (2/481) specimens from HIV-positive cases, less (p<0.0001) than 4.7% specimens from HIV-negative cases. Specifically, 1 HIV-positive case with Stegen-Toledo score >6 (indicating highly-probable TB) had positive cultures only with the MODS assay for both gastric aspirates. Consequently, cases were less likely (p = 0.009) to be microscopy or culture-positive if they were HIV-positive (1.2%, 1/81) than HIV-negative (11%, 22/209).

### Test speed

We have reported culture speed previously [18]. PCR and microscopy results were available within 24 hours whereas culture took at least 5 days. Negative culture results were issued after 30 days for MODS and 42 days for Lowenstein-Jensen.

### PCR versus culture results (Tables 2 and 3)

Specimens were more likely to be PCR-positive than culture-positive for all 4 participant groups (all P≤0.01).

**Table 3:** PCR and culture results.

	Test	Positive specimens	44 HIV-positive cases with ST>4				44 HIV-positive cases vs 35 HIV-positive well-controls	44 HIV-positive cases vs 209 HIV-negative cases
Children			%	95%CI	n	N	P-value	P-value
	PCR	Any	30%	13–31	13	44	NA	0.5
	Microscopy & culture	Any	2.3%	0.0–3.6	1	44	NA	0.01
	P-value PCR vs microscopy & culture		<0.0001				NA	NA
Specimens	PCR	NPA	8.0%	3.3–16	7	88	0.9	0.7
		GA	8.0%	3.3–16	7	88	ND	0.6
		Stool	5.8%	1.9–13	5	86	1.0	0.7
		NPA+GA+Stool	7.3%	4.4–11	19	262	0.9	1.0
	Microscopy & culture	NPA	0.0%	0.0–4.2	0	88	1.0	0.004
		GA	2.3%	0.0–2.9	2	88	ND	0.002
		Stool	0.0%	0.0–4.3	0	86	1.0	0.2
		NPA+GA+Stool	0.76%	0.0–1.0	2	262	0.5	<0.001
	P-value PCR vs microscopy & culture		<0.001				NA	NA

The percentage and number of children and specimens from children with at least one positive PCR or culture.

The Stegen-Toledo cut-off for inclusion in this analysis was identical for HIV-positive and HIV-negative children (see Methods).

Note: TB = tuberculosis; NPA = nasopharyngeal aspirate; GA = gastric aspirate; NA = not applicable; ND = not done; ST = the Stegen-Toledo score assessing the likelihood of TB disease (see Methods).

**Supplementary analysis (Table 3)** considering only cases with Stegen-Toledo score >4 (i.e. excluding HIV-positive cases with Stegen-Toledo score = 4 so inclusion criteria were identical for HIV-positive and HIV-negative cases) had little effect and significant differences between groups were maintained except the P-value for recent weight loss increased to 0.075.

### Microscopy and culture test efficiency (Fig 3)

Microscopy detected 22% (95%CI 7.4–44, 5/23) of culture-proven TB. Lowenstein-Jensen culture detected 52% (95%CI 31–73, 12/23) of TB diagnoses. MODS (without Lowenstein-Jensen) detected 91% (95%CI 72–99, 21/23) culture–proven TB, but MODS together with Lowenstein-Jensen culture had to be done to detect 100% (23/23) culture-proven TB.

**Fig 3:**
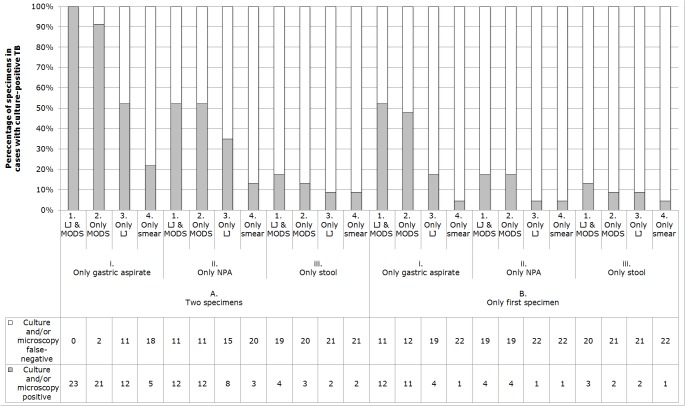
Tuberculosis (TB) microscopy and culture test results for specimens from the 23 cases with culture-positive TB. All subjects had two specimens of each type analyzed for TB by the methods shown. The graph shows the proportion (and the data table the number) of patients who had positive results in (A.) at least one of their two specimens and (B.) who had positive results in the first specimen of their two specimens, with separate bars indicating results for stool, nasopharyngeal aspirate (NPA) or gastric aspirate specimens tested only by auramine microscopy (smear), Lowenstein-Jensen (LJ) solid culture, or microscopic-observation drug-susceptibility (MODS) liquid culture [e.g. the first bar demonstrates that all 23 cases would have been diagnosed if only (1.) LJ and MODS culture had been performed (i.e. without microscopy) on (A.) duplicate gastric aspirate specimens].

### Specimen efficiency (Fig 3)

Considering all microscopy and culture-positive participants, 100% (23/23) had at least one culture-positive gastric aspirate, whereas microscopy and culture testing of nasopharyngeal aspirates confirmed 52% (95%CI 31–73, 12/23) and stool specimens confirmed 17% (95%CI 5.0–39, 4/23) of culture-positive cases without contributing any additional TB diagnoses. Testing only the first gastric aspirate specimen (instead of duplicate gastric aspirates) would have caused 48% (95%CI 27–69, 11/23) of the culture-positive cases to be missed.

### Number of tests needed per TB diagnosis (Fig 4)

1458 microscopy and culture tests were done to detect each HIV-positive culture-positive case, whereas 171 microscopy and culture tests were done to detect each HIV-negative culture-positive case. If gastric aspirates had been the only specimen tested then all of the culture-positive cases would still have been detected and the number of microscopy and culture tests done to detect each culture-positive HIV-positive case would have been 486, whereas 57 would have been done to detect each HIV-negative case.

**Fig 4:**
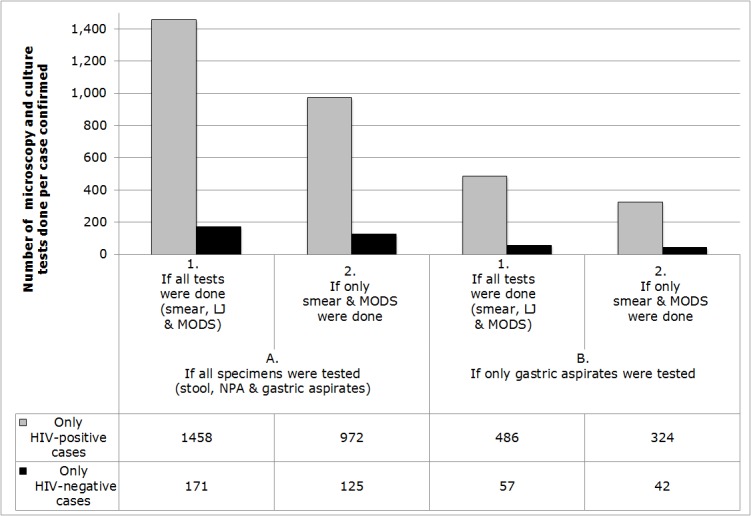
The number of tuberculosis (TB) microscopy and culture tests done for each case confirmed. A. All cases had duplicate stool, nasopharyngeal aspirate (NPA) and gastric aspirate specimens tested for tuberculosis (TB) by auramine microscopy (smear), Lowenstein-Jensen (LJ) solid culture, and microscopic-observation drug-susceptibility (MODS) liquid culture [e.g. the left-most gray bar indicates that in this study 1458 tests (1. smear, LJ and MODS) were done (on A. stool, NPA and gastric aspirates) per HIV-positive case diagnosed; whereas the right-most black bar indicates that in this study if only (2.) smear and MODS testing had been done on only (B.) duplicate gastric aspirates then 42 tests would have been done per HIV-negative case diagnosed].

## Discussion

These data demonstrate the challenges of diagnosing TB in HIV-positive children and the advantages and disadvantages of diverse strategies, specimens and tests. Laboratory results from children with culture-proven and clinically suspected TB, were compared to results from well-controls, strengthening the study by providing novel insights to the overall performance of these tests within the HIV-infected population, which is lacking from most previous studies of pediatric TB diagnosis.

Although TB diagnosis by same-day PCR testing seemed desirable, most culture-positive cases and specimens had false-negative PCR. Furthermore, PCR in well-controls was prone to false-positives, as in previous studies [16, 17]. Consequently, PCR-positives were slightly more likely in well-controls then suspected TB cases. Thus in HIV-positive children with suspected TB in this setting, the in-house PCR did not provide clinically relevant results and cannot be recommended.

The recently implemented GeneXpert MTB/RIF commercial TB PCR test uses disposable cartridges that may reduce the risk of cross-contamination causing false-positives [23, 24]. However, a GeneXpert meta-analysis reported a pooled specificity of 98% (95%CI 97–99%), similar to the in-house PCR specificity of 97.4% (95%CI 96.3–98.6) that we observed, although specificity estimates are difficult to compare between studies with different inclusion criteria; positives may reflect TB disease that was missed by other tests. These findings emphasize the importance of false-positive PCR results, the need to include control participants when evaluating diagnostic tests and demonstrates that even rare false-positives may out-number true-positives in pediatric TB diagnosis because of the low diagnostic yield of TB diagnostic tests in children.

The inadequacy of these PCR results only became apparent because well-controls were included in similar numbers to cases, in contrast to many previous studies of pediatric TB diagnostics. Recent guidelines emphasized the importance of well-controls in early assessment of new diagnostics and our findings here suggest they may also be important for late, clinical assessment of pediatric TB diagnosis because it is difficult to determine which symptomatic children have TB disease [25]. We noted a significantly higher false-positive PCR rate in HIV-positive than HIV-negative controls and this association should be studied in future research. Although the low prevalence of HIV infection in Peru [26] necessitated recruitment of some controls who had recent TB contact, this did not explain these false-positive PCR that occurred significantly more frequently in controls who had no known TB contact. All PCR-positive well-controls were followed up for at least 1 year and all remained free from clinically apparent TB, implying that these false-positive PCR results were not explained by early or sub-clinical TB.

PCR sensitivity was difficult to assess because false-positives were more common than true-positives. However in HIV-positive cases, >92% of specimens were PCR-negative from children with culture-positive TB, highly probable and probable TB. Similarly, frequent PCR false-negative results in culture-confirmed pediatric TB were reported in previous research assessing GeneXpert testing of induced sputa in South Africa [27] and in high-risk children in our previous research in Peru [16]. Thus, some culture-positive cases are missed using molecular methods, probably as a result of the paucibacillary nature of pediatric TB [16, 23]. In contrast to some reports from Africa, we found that TB recovery from HIV-positive patients with suspected pulmonary TB in this Peruvian population was much less common than in HIV-negative cases. Indicators of pulmonary TB used to calculate clinical scores may be less specific for TB among HIV-infected children. This implies that many HIV-infected participants may have had other pulmonary infections instead of TB. Including a third gastric aspirate specimen and possibly induced sputa in addition to gastric aspirates may increase diagnostic yield in future work [13, 27].

In contrast to the poor specificity of PCR, microscopy and culture testing were reliable because there were no false-positive results amongst >2000 tests performed blindly and concurrently on specimens from well-controls. This validates the reliability of these tests including the MODS assay, and contrasts with reports of false-positive microscopy and culture test results in some settings [28, 29]. The absence of false-positive microscopy and culture tests in the present study may be partly explained by the infrequent rate of culture-positivity in specimens from cases processed concurrently with the specimens from well-controls.

In HIV-positive children with suspected TB, diagnostic yield was so low that 1458 microscopy and culture tests were done per case confirmed. We identified combined same-day microscopy plus MODS testing (that took 30 days) of duplicate gastric aspirate specimens as the most efficient pediatric TB testing strategy, but even this approach required 324 microscopy and culture tests to confirm each HIV-positive case, which is unlikely to be cost-effective in most settings. Even in specimens from children with culture-positive TB, most specimens were negative by all tests. Thus, negative microscopy and culture tests could not rule out TB. Almost half of microscopy and culture test-positive results were missed by the first specimen and occurred only in second specimens. Nasopharyngeal aspirates and stool specimens did not contribute any additional diagnoses to those found by testing gastric aspirates. In most resource-constrained settings the only TB laboratory tests available are acid-fast microscopy and in some settings culture on egg-based medium that only detected approximately half of the cases detected by the MODS assay.

Limitations inherent to most studies of pediatric TB diagnosis include the lack of a gold standard test and reliance on well-controls because it would be difficult to be sure whether symptomatic controls had versus did not have TB. Without a sensitive gold standard test, some subjects with negative microscopy and culture tests and positive PCR may have pulmonary TB. Data concerning treatment given, response and outcome were not included in this study because they would have been influenced by the results of the tests under evaluation. Specific limitations to the present study include the imperfect age and sex matching and limited numbers of HIV-positive well controls that resulted from HIV infection being infrequent in the study population [26], the use of clinical definitions that we developed prior to recent recommendations for standardization [25], and the fact that we did not study alternate diagnoses that may have been made by clinical practitioners.

In conclusion, in HIV-positive children with suspected TB, PCR was so prone to false-positives that positive PCR results were as likely in specimens from well-controls as from cases, so the PCR was not clinically informative. This was only apparent thanks to, and hence demonstrates the importance of including well-control participants when evaluating diagnostic tests. In contrast, microscopy and culture TB testing was reliable because there were no false-positive results amongst microscopy and culture tests from well-controls. However, microscopy and culture testing generally took weeks, limiting impact on TB treatment decisions. Furthermore, microscopy and culture testing had such low diagnostic yield that it was inefficient for detecting TB and could not rule out TB, especially in HIV-positive children. The most efficient testing strategy would have been testing duplicate gastric aspirates with the MODS assay because others tests and specimens added little. However, universal extensive, state-of-the-art TB laboratory testing only rarely significantly contributed to the clinical assessment and care of children with suspected TB.

## Supporting Information

S1 Dataset The dataset for this study is included as supplemental information along with a legend defining specific variables. (XLSX)
